# The characteristics and prognostic significance of the SET-CAN/NUP214 fusion gene in hematological malignancies: A systematic review

**DOI:** 10.1097/MD.0000000000029294

**Published:** 2022-07-29

**Authors:** Jing Wang, Qian-ru Zhan, Xiao-xuan Lu, Li-jun Zhang, Xiao-xue Wang, He-yang Zhang

**Affiliations:** Department of Hematology, The First Hospital of China Medical University, Shenyang 110001, China.

**Keywords:** acute lymphoblastic leukemia, hematological malignancies, prognosis, HSCT, SET-CAN/NUP214 fusion gene

## Abstract

**Background:**

The SET-CAN/NUP214 fusion gene resulting from chromosomal del(9)(q34.11q34.13) or t(9;9) (q34;q34) has been found in T-cell acute lymphoblastic leukemia (T-ALL), B-cell acute lymphoblastic leukemia (B-ALL), acute myeloid leukemia (AML) and myeloid sarcoma (MS). Furthermore, the SET-CAN/NUP214 fusion gene has been found in the T-ALL cell line LOUCY and the AML line MEGAL. The common features of these cases are insensitivity to chemotherapy and poor prognosis. We reviewed the characteristics and prognostic significance of the SET-CAN/NUP214 fusion gene in hematological malignancies.

**Methods:**

This systematic literature search was conducted using the PubMed, Web of Science, Embase, and Cochrane Library databases. With the inclusion and exclusion criteria, we summarized all of the papers and performed a statistical analyses.

**Results:**

In general, the SET-CAN/NUP214 fusion gene is very rare in adult acute leukemia, more frequently found in T-ALL than in other types of leukemia, and more often in males. Flow cytometry data indicated that the markers CD34, CD33, CD13, and CD7 were common in SET-CAN/NUP214 positive acute leukemia, including ALL. Fluorescence in situ hybridization and arrays are important methods for detecting the fusion gene in newly diagnosed patients and can detect chromosomal del(9)(q34) will be detected. The chromosomal karyotype may be normal or complex, and, in terms of survival analysis, transplantation results in a better prognosis than chemotherapy alone.

**Conclusions and implications of key findings:**

The presence of SET-CAN/NUP214 fusion gene may be a Minimal Residual Disease of early recurrence, and it might be a poor indicator of outcome.

**Limitations:**

The mechanism, clinical characteristics, therapy and prognosis of the SET-CAN/NUP214 fusion gene in hematological malignancies require further research.

## 1. Introduction

Rapid advances in cytogenetics and molecular genetics have played an important role in the detection of hematological malignancies in accordance with the 2016 World Health Organization classification of myeloid neoplasms and acute leukemia.^[1]^ These methods loosely contribute to the determination of differences in characteristics, treatment strategies, and prognosis of the acute leukemia. The wide use of fluorescence in situ hybridization (FISH) has revealed some more chromosomal translocations with submicroscopic deletions, including del(1)(p32) (TAL1 gene deletion), del(4)(q12) (FIP1L1-PDGFRA fusion gene), cryptic 11q23 deletions (MLL-LARG or MLL-CBL rearrangement), and t(9;9)(q34;q34) or del(9)(q34.11q34.13) (SET-CAN rearrangement).^[2,3]^The SET gene, also known as TAF1, encodes TAF1-α and TAF1-β proteins, and the majority of SET-CAN fusion gene present the TAF1-β isoform. TAF1-β protein is an inhibitor-2 of protein phosphatase-2A and acts as a histone acetyltransferase. This protein is located in the nucleus and performs histone binding and chromatin remodeling activities. The CAN gene, also known as nucleoporin (NUP) 214, is a component of the nuclear pore complex and plays a key role in the nuclear export of proteins and mRNAs. Chromosomal translocations result in the joining of the NUP214 C-terminus with SET or DEK, which forms the SET-NUP214 or DEK-NUP214 fusion proteins. Both of them play an important role in transcriptional regulation.^[4]^

In this review, we mainly discuss the SET-CAN/NUP214 fusion gene. The SET-CAN fusion gene has been found in the T-cell acute lymphoblastic leukemia (T-ALL) cell line LOUCY and acute myeloid leukemia (AML) line MEGAL. It occurs as del(9)(q34.11q34.13) or t(9;9)(q34;q34),^[4,5]^ which was firstly reported in a case of acute undifferentiated leukemia (AUL) in 1992.^[6]^ Since then, several additional cases have been reported. The SET-CAN fusion gene has been found in T-cell acute lymphoblastic leukemia (T-ALL), B-cell acute lymphoblastic leukemia (B-ALL), acute myeloid leukemia (AML) and myeloid sarcoma (MS).^[2,7–9]^ It is reported more frequently in T-ALL, which accounts for approximately about 50% of the cases. The common features of these cases are insensitivity to chemotherapy and poor prognosis,^[9]^ however, the mechanism is still unclear. It is considered that hematopoietic stem cell transplantation (HSCT) may improve the outcome,^[7]^ at the same time, detection of the SET-CAN fusion gene in minimal residual disease (MRD) may be a prognostic indicator of early disease recurrence after HSCT. New therapies, such as chimeric antigen receptor T (CAR-T) cell therapy, require further research.^[10]^ In this systematic review, we summarized the characteristics and prognostic significance of the SET-CAN fusion gene in hematological malignancies.

## 2. Materials and methods

This systematic literature search was independently performed by 2 authors (Wang and Zhang) using the databases PubMed, Web of Science, Embase and Cochrane Library databases. There was no time limit for the literature search and the language was restricted to English. We used the keyword strings shown in Table 1, along with the appropriate MeSH- terms and original words. All eligible studies were considered for this review in order to conduct an exhaustive literature search.

**Table 1:** The string of key words.

Fusion gene	SET-CAN OR SET-NUP214 OR SET-CAN protein OR SET-NUP214 protein OR TAF-1-CAN
AND	
Disease	Leukemia OR acute lymphoblasic leukemia OR acute myeloid leukemia OR myeloid sarcoma OR hematological maligancies OR acute undifferentiated leukemia OR ALL OR AML OR MS OR AUL

The other 2 authors (Lu and Zhang) used the following inclusion and exclusion criteria for the literature search by scanning the full text. The inclusion criteria were as follows: (1) any type of adult hematological malignancy was involved; (2) expression of the SET-CAN(SET-NUP214) fusion gene; (3) complete characteristics, treatment and outcomes. The exclusion criteria were as follows: (1) nonhematological diseases; (2) lack of complete and detailed information; (3) hematological malignancy in children. Data extraction and quality assessment were based on information from published studies, conference abstracts, protocols and contact with study authors. If there was a disagreement between the 2 authors, a consensus was reached after discussion with the other 2 authors (Wang and Zhang). We summarized all of the papers and made a statistical analysis using the software of SPSS23.0 software. The outcome was considered to be statistically significant if *P* < 0.05. The Kaplan-Meier survival curves were used for the survival analysis.

## 3. Results and discussion

**Figure 1.**
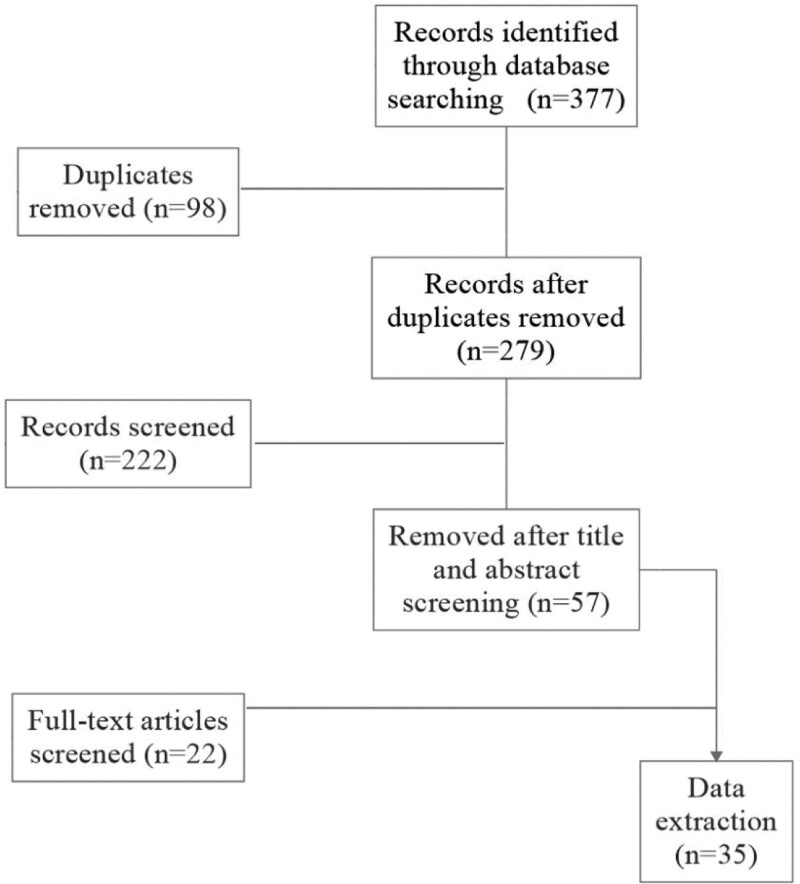
Flow chart of study selection from literature search.

A total of 377 papers were retrieved after a systematic literature search. Ninety-eight papers were removed because of duplication of the content. According to the inclusion and exclusion criteria mentioned above, 222 articles were excluded after reading the literature abstracts. Therefore, 57 papers were identified by full-text screenings. Finally, there were 35 articles that met the inclusion criteria contained for the systematic review. The entire retrieval process was shown in Figure 1.

There have been no prospective clinical studies of patients with the SET-CAN/NUP214 fusion gene because the gene is not well-known. Most of the articles were case reports and others were mainly about the mechanism and research progress of the SET-CAN fusion gene. We analyzed the results separately according to the different types of leukemia.

### 3.1. ALL

ALL is a hematological malignancy characterized by chromosomal abnormalities and genetic alterations that affect the B-lineage, T-lineage, and NK-lineage. The incidence rates of ALL are 85%, 10%–15%, and < 1% for B, T, and NK lineages,^[11]^ respectively.

T-ALL is characterized by the clonal proliferation of T-line progenitor cells rather than normal hematopoietic cells found in the bone marrow. The long-term survival rate for adult ALL is only 30%–50%.^[12]^ Therefore, there are still substantial challenges that remain for improving the treatment and prognosis of ALL cases.

The SET-CAN/NUP214 fusion gene has been reported in some cases of T-ALL, both in children and adults. According to the report of French Group for research on Adult Acute Lymphoblastic Leukemia (GRAALL) 2003 and 2005 trials reported in 2014, the incidence of SET-CAN/NUP214 positive fusion gene was 6% in T-ALL patients.^[13]^ In a study by Gorello et al, 7 out of 152 adult T-ALL patients (4.6%) expressed the SET-CAN fusion gene.^[14]^ The results of flow cytometric analysis of SET-CAN-positive patients with T-ALL showed that they not only expressed T lymphocyte antigen, but also myeloid antigens, especially CD33 and CD13.^[15]^ This suggests that the tumor cells of these patients may be in the early stage of T lymphocyte development. The deletion and ectopic formation of small fragments on chromosome 9 are not detectable by conventional cytogenetic methods. Therefore, the use of FISH and array is important in such cases. The del(9)(q34) can be detected in all of the patients and the chromosomal karyotype may be normal or complex. Studies have shown that such patients are resistant to glucocorticoids and traditional chemotherapy. In the report of T Ichijo, 2008, a potential mechanism of glucocorticoid resistance might be that the SET-CAN/NUP214 fusion protein is constitutively co-precipitated with glucocorticoid response elements. And the fusion protein suppresses glucocorticoid receptor transcriptional activity and histone acetylation.^[16]^ According to a report from Yang et al 2020,^[7]^ 3 SET-CAN/NUP214 positive patients with T-ALL patients had been reported. They were resistant to high-dose glucocorticoid-based chemotherapy and died of infection. These authors suggested that inhibition of histone H3 acetylation may be the underlying mechanism of glucocorticoid resistance and asparaginase combined with CLAG chemotherapy (cladribine, cytarabine, and granulocyte-colony stimulating factor) may be a potential treatment. However, in the report of Abdelali et al at 2014, published in Blood, concluded that SET-CAN/NUP214 was strongly associated with corticosteroid and chemotherapy resistance, but did not negatively affect clinical outcomes. Compared with SET-CAN/NUP214 negative patients, SET-CAN/NUP214 positive patients showed a significantly higher rate of corticosteroid resistance (91% vs 44%; *P* = .003) and chemotherapy resistance (100% vs 44%; *P* = .0001). Interestingly, the event-free survival (EFS) and overall survival (OS) at 3 years of the SET-NUP214 positive patients were not significantly different from those of SET-NUP214 negative patients (45% vs 59%; *P* =0.52 for EFS and 73% vs 68%; *P* = .86 for OS). Early hematopoietic stem cell transplantation is necessary after complete remission of the disease. In a report published by Gao M G in 2020,^[17]^ the SET-CAN/NUP214 fusion gene was shown to be a sensitive and specific MRD indicator for the acute leukemia patients received allogeneic hematopoietic stem cell transplantation(allo-HSCT). Patients who were positive for the SET-CAN/NUP214 fusion gene after HSCT would had poor outcomes. Further studies are needed to evaluate the incidence of SET-CAN/NUP214 rearrangement and treatment response in patients with T-ALL, as well as the prognosis of these patients.

**Figure 2.**
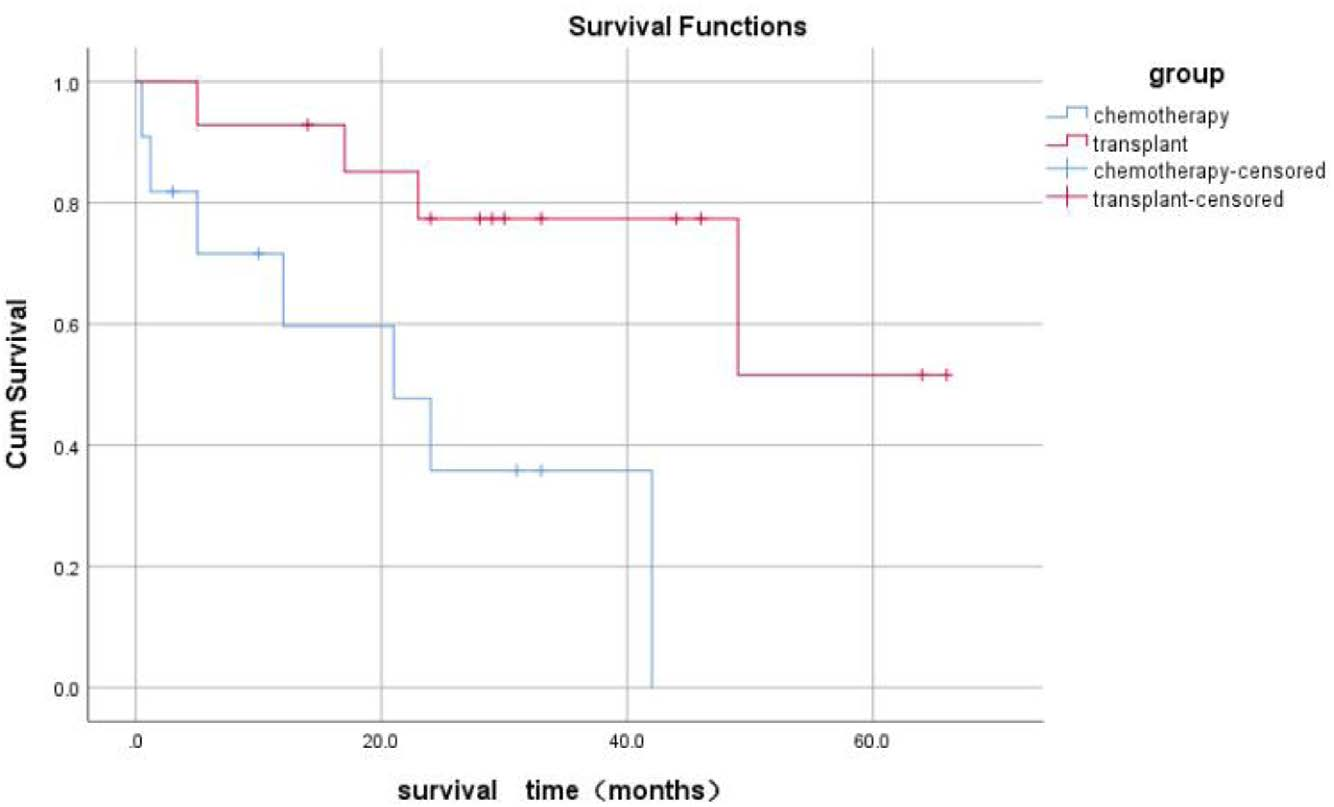
The Kaplan-Meier survival curve of SET-CAN/NUP214 positive T-ALL patients.

Here we analyzed the characteristics of SET-CAN/NUP214 positive T-ALL in adults. In this systematic review, there were 8 papers reported SET-CAN fusion gene in T-ALL, including 30 patients. Five papers were excluded because of incomplete information. The detailed information was shown in Table 2. In this review, the mean age of T-ALL patients with the SET-CAN/NUP214 fusion gene is 33 years old. Among the 30 patients, 6 were females and 24 were males, which suggests that the incidence of ALL with the SET-CAN fusion gene is more likely to develop in male. The most common cell surface markers were CD7, which was positive in 21 of 23 patients (91.3%), and CD34 in 16/23 (69.5%) patients. CD33, which is mainly expressed in AML, was found in 15 of the 23 patients with detailed flow cytometric descriptions, accounting for 65.2%, as well as 4 patients with CD13 expression. In the total 25 valid cases, 11 patients underwent chemotherapy and 14 received transplants, with 7 and 4 deaths each, respectively. The mean survival was 22.5 months (95% confidence interval [CI], 11.3–33.7) in the chemotherapy group and 50.1 months (% CI, 37.7–62.6) in the transplant group. The mean survival time in the transplant group was almost twice that in the chemotherapy group, indicating that chemotherapy treatment alone was not sufficient for the patients with SET-CAN/NUP214 fusion gene. The results showed that the difference between the 2 groups was statistically significant (*χ*^2^ = 6.761, *P* = 0.009). The Kaplan-Meier survival curve is shown in Figure 2. It is clear that HSCT can significantly extend overall survival (OS).

**Table 2:** The characteristics of adult SET-CAN T-ALL cases.

Ref.	Sex	Age(y)	WBC(×10^9^/L)	Immunophenotype positive	Chromosome	FISH	Treatment	Outcome
Yang Q. 2020^7^	Male	26	12.3	CD7, CD99	46,XY, del(11)(q13), del(13)(q14), inv(16)(p13.3q23)	NR	VICP	Dead; +15 days
	Male	51	109.1	CD7, CD33, CD99, CD10	NR	NR	VICP, mitoxantrone, etoposide, cytarabine	Dead; +37 days
	Male	37	131.5	CD7, CD99, CD38, CD34, CD33, HLA-DR	5,XY, der(17;19)(q10;q10) 46,XY	NR	CALGB9111, CLAG,	Alive; >10 months
Zhang H.Y. 2020^9^	Male	21	37.16	CD3, CD99, CD4	46,XY	del(9)(q34)/ABL1	VICP*2, Hyper-CVAD B, MTX, Cladribine,decitabine	Achieved CR after the first cycle of chemotherapy. Continue consolidation therapy and allo-HSCT, CR, SET-CAN (-), alive 14 months
Lee S.G. 2011^2^	Male	28	37.3	CD5, CD7, CD33, CD34	47,XY, del(1)(p13p22), del(6)(q13q21), del(9)(q12), del(11)(q13), −12, add(15)(p11.2), del(16)(q22), +19, +mar[3]/46, XY [17]	NR	prednisolone, vincristine, L-asparaginase, daunomycin, cytarabine, andmethotrexate,	CR, SET-NUP214 fusion transcript+. The patient is scheduled to receive HSCT from an unrelated donor.
Lee E.Y. 2012^18^	Female	43	60.6	CD3, CD5, CD7, CD13, CD33, CD34	46,XX,dup(1) (p22p36.1)	del(9)(q34)/ABL1	NR	NR
Chae 2011^15^	Female	55	24.43	CD33, CD34, CD13,CD7, cy-CD3	47,XX,del(11)(q22q23), del(12)(p13),+14	del(9q34)/ABLl	NR	Relapse 31 months
	Male	32	18.04	CD33, CD34, CD13,CD7, CD5, cy-CD3	46,XY,del(13)(q12q14)	del(9)(q34)/ABL1	NR	Relapse and death, 42 months
	Male	32	39.06	CD33, CD34, HLA-DR,CD7, cy-CD3	46,XY,del(6)(q21q23),del(12)(p11.2)	del(9)(q34)/ABL1	NR	Relapse and death, 21months
	Female	20	5.07	CD33, CD34, CD7,CD5, CD8, Cy-CD3	46,XX,+del(3)(q11.2)del(12)(p13),-13,add(17)(p.11.2)	del(9)(q34)/ABL1	NR	HSCT, 41 months
Ben Abdelali 2014^13^	Male	34	30.4	CD34、CD33、CD7、cCD3 (ETP-ALL)	46, XY, t(3;10)(q?;q?)[20]	NR	GRAALL trail	CR, relapse, SCT, died 49 months
	Female	37	8.6	CD34、CD7、cCD3 (ETP-ALL)	46,XX,t(4;16)(q2?6;q23)[30]	NR	GRAALL trail	CR, SCT, alive 64 months
	Male	29	10.1	CD34、CD13、CD33、CD7、cCD3 (ETP-ALL)	46,XY,del(6)(q14q24),del(11)(q21),del(12)(p12)[9]/46, XY[3] ### del(9)(q34.11q34.13) ### NR	NR	GRAALL trail	CR, relapse, SCT, alive 44 months
	Male	41	18.4	CD34、CD33、CD7、cCD3 (ETP-ALL)	47,XY,+4[15]	NR	GRAALL trail	CR, SCT, alive 46 months
	Male	23	604.4	CD7、cCD3	46,XY[31]	NR	GRAALL trail	Died 5 months
	Male	30	24.9	CD7、cCD3	46,XY[21]	NR	GRAALL trail	CR, SCT, relapse, CR, alive 66 months
	Male	36	181.8	CD34、CD33、CD7、cCD3	46,XY,add(5)(q22),del(12)(p11p13)[2]/46,XY,der(5)t(5;12)(q11.2;p13),del(12)(p11p13),der(12)t(5;12)(q11.2;p13)add(5)(q22)[2]/46,XY[16]	NR	GRAALL trail	CR, SCT, alive 24 months
	Male	45	50.8	CD7、cCD3	46,XY,del(5)(q?q?)[7]/46,XY,del(13)(q12q14),inv(14)(q11q32),del(16)(p12p13.3)[5]/46,XY[5]	NR	GRAALL trail	CR, alive 33 months
	Male	38	2.8	CD34、CD33、CD7、cCD3 (ETP-ALL)	88,XX,-Y,-Y,[4n],add(2)(q24),+4,-5,-5,add(5)(q?35),-7,-9,add(9)(p21),del(9)(q11q12),+10,del(12)(p13)x2,-17x2,+2mar[cp7]/77~89,sl,+Y,+Y,-add(9),-del(9),+9,+9,+1~2mar[cp3]/78~88,sdl1,-9,add(15)(p11)[cp6]/46,XY[1]	NR	GRAALL trail	SCT, died 9 months
	Male	28	41.8	CD34、CD33、CD7、cCD3	46,XY,del(5)(q31q35),del(6)(q?12q?16),del(7)(q34),del(12)(p12),del(16)(q2?)[29]/47,idem,del(11q),+mar[6]/46,XY[3]	NR	GRAALL trail	CR, SCT, alive 30 months
	Male	20	30.9	CD7、cCD3	48,XY,+21,+21 [5]/ 46,XY [25]	NR	GRAALL trail	CR, SCT, alive 28 months
Prokopiou C 2015^19^	Female	48	NR	CD7+, CD5 dim, sCD3-, cCD3+, CD4-, CD8-, CD34+, HLA-DR+, CD117+, MPO+	del (17)(q11.2), del(6)(q16.1-q21) and del(12)(p12.1-13.1)	NR	combination chemotherapy	ASCT from her fully matched sibling, relapsed 1 year after ASCT, died during induction therapy
	Male	45	NR	CD7+, CD38+, CD34+, CD3+, CD4-, CD8-,CD33+, CD1a-	del (17)(q11.2), del(6)(q16.1-q21) and del(12)(p12.1-13.1)	NR	combination chemotherapy	ASCT from a fully matched unrelated donor, died 6 months after ASCT
Gorello 2010^14^	Male	38	24	Pre-T	46,XY[15]	del(9)(q34)/ABL1	NR	CR, ASCT, alive +29 months
	Male	19	3.28	Pre-T	46,XY[15]	del(6)(q16)/GRIK2 del(9)(q34)/ABL1 del(12p)/ETV6	NR	CR, SCT, relapse, Cord blood transplant, died +23 months
	Male	47	NR	Cortical	NR	del(9)(q34)/ABL1	NR	Refused treatment
	Female	27	NR	Pre-t	NR	del(9)(p21)/CDKN2A-B del(9)(q34)/ABL1 del(11)(p13)/LMO2 del(11)(q14)/CALM	NR	Resistant died +12 months
	Male	19	NR	Pro-T	NR	del(9)(q34)/ABL1 del(11)(p13)/LMO2 del(11)(q14)/CALM del(12)(p13)/ETV6	NR	CR, alive +3 months
	Male	18	NR	Pre-T	NR	del(9)(q34)/ABL1 del(5)(q35)/TLX3	NR	CR, relapse, died +24 months
	Male	23	NR	Pre-T	46,XY[12]	del(9)(q34)/ABL1	NR	CR, relapse, ASCT, died +17 months

To date, only 2 cases of SET-CAN/NUP214 positive B-ALL have been reported (Table 3). The first was published in 2010 by Nowak NJ, et al,^[20]^who described an adult normal karyotype precursor B-ALL. Through array and FISH, del(9)(q34) was found. Furthermore, these authors determined that the SET-CAN/NUP214 fusion gene resulted in upregulation of the HOXA gene cluster, which was also reported in another study.^[21]^ Zhu H, et al had reported the second case of SET-CAN fusion gene in B-ALL.^[8]^ The patient was a 19- year-old male with a complex karyotype abnormality. The immunophenotypic analysis revealed not only B-ALL phenotype was positive, but also T-ALL phenotype was expressed, including CD34, CD33, CD13 and CD7. The patient was resistant to chemotherapy with the failure of remission after the Induction chemotherapy. Due to the limited number of cases, the situation of SET-CAN/NUP214 positive B-ALL will require further evaluation to understand, including the frequency of SET-NUP214 rearrangement, its prognostic significance, and certain clinical features. Additional data will help define a new specific acute leukemia subtype and guide its treatment.

**Table 3:** The characteristics of adult SET-CAN B-ALL cases

Ref.	Sex	Age (y)	WBC(×10^9^/L)	Immunophenotype positive	Chromosome	FISH	Treatment	Outcome
Zhu 2016^8^	Male	19	217	HLA-DR+, CD34+, CD38 +, CD58+, cytoplasmic (c) CD79a+, CD19+ (dim), CD22+ (dim), CD33+, CD13+, CD7+, CD11b+, CD10-, CD117-, cCD3-, CD4-, CD8-,CD20-, CD25-, CD103-,	56,XY,+6,+8,+12,+13,+15,+19,+20,+21,+21,+mar(1)/45-49and 48,XY, +12,+15,+16,i(17)(q10), +21,+22,+mar2(cp5)/46,XY (4).	NR	Cyclophosphamide Vindesine Daunorubicin Prednisone	–
Nowak N.J. 2010^20^	Female	42	NR	NR	NR	del(9)(q34)	NR	NR

### 3.2. Other types of leukemia with the SET-CAN/NUP214 fusion gene

While most of the SET-CAN/NUP214 fusion gene is present in ALL, it had also been reported in other types of leukemia, including AML, AUL, and MS (Table 4). Here we collected 6 studies published in English that included 7 patients, 3 were diagnosed with AML, 2 with AUL (2/7), 1 with MS (1/7), and 1 presented with mixed phenotype acute leukemia (MPAL, 1/7).^[3,6,9,10,22,23]^ Two other cases diagnosed with AML were excluded because of a lack detailed information.^[24]^ The MS patient was female, and the 6 leukemia patients were male, which was similar to the female:male ratio observed for T-ALLs with the SET-CAN/NUP214 fusion gene. The median age was 32.1 years old (19–46), young and middle age. The mean account of white blood cell was 18.0 × 10^9^/L (0.56–53 × 10^9^/L). Immunophenotypic analysis revealed that the cell surface markers CD33, CD34, CD7, and CD13 occurred in 100%, 66.7% (4/6), 83.3% (5/6), and 33.3% (2/6) of the AML, AUL, MS, and MPAL cases, respectively, and 71.4% (5/7) patients had a normal chromosomal karyotype. Survival analysis was not performed due to the small number of reported cases. According to the report of Kandilci, published in 2004, the mechanism of SET-CAN/NUP214 fusion gene in AUL was associated with the apoptosis in the U937 cell line.^[25]^The SET/TAF-Iβ-CAN fusion protein in AUL binds the nuclear export protein hCRM1, disorganizes nuclear export, causes cell cycle arrest at S phase, and partially blocks vitamin D3-induced differentiation. In the case of Rosati et al, they firstly found TAF-1α-CAN fusion gene, but the influence of this gene remained unknown.^[23]^

**Table 4:** The characteristics of adult SET-CAN AML and AUL cases.

Ref.	Diagnosis	Sex	Age (y)	WBC(×10^9^/L)	Immunophenotype positive	Chromosome	FISH	Treatment	Outcome
Zhang H.Y. 2020^9^	AML	Male	24	11.41	HLA- DR, CD7, CD33, CD34, CD11b, CD123, CD64, CD13	46,XY	NR	Daunorubicin, Cytarabine	Alive for 8 months
	MS	Female	32	4.15	CD7(+), CD33(+), CD43(+), CD99(+), CD4(+), CD117, CD8	46,XX	NR	Idarubicin, Cytarabine Homoharringtonine	Myelosoppression with a rapidly increased pericardial effusion
Jeong I.H. 2019^22^	AML-M1	Male	46	17.1	CD33, CD7, CD34, CD71	59–90, XXXY, -1, −2, -5, -7, -7, -10, -13, -13, -16, -17, -18, -21[cp23]	del(9)(q34)/ABL1	idarubicin, cytosine, arabinoside	CR and MR, still alive for 8 months
Rosati 2007^23^	AML-M4	Male	35	40	positive for myeloperoxidase, CD34, CD33, CD13, CD45, CD66b, CD15 and CD11b antigens	46,XY	del(9)(q34)/ABL1	Daunorubicin and cytosine arabinoside	CR,HSCT from his HLA-identical brother 4 months after diagnosis
Kim J. 2010^3^	AUL	Male	40	53	CD7, CD33, CD117, CD38, cCD3, cCD22, cCD79a	46,XY[20]	del(9)(q34)/ABL1	Cytosine, Arabinoside, Idarubicin	CR, alive 7 months
Vonlindern 1992^6^	AUL	Male	19	NR	NR	46,XY	NR	NR	NR
Li M.Y. 2020^10^	MPAL	Male	29	0.56	CD7, CD34, HLA-DR, CD10, CD19, CD33, CD117, cCD79a, cCD3.	46,XY, add(6)t(?1;6)(?p31;p24), del (16)(?q11)[6]/46,XY[4]	NR	idarubicin, vincristine, and dexamethasone, Hyper CVAD-B、Hyper CVAD-A、HSCT、 and CAR-T	relapsed within 6 months after HSCT, relapsed after 2 years of first CAR-T; then second CAR-T, after 8 months,still alive; totaly alive >42 months

One patient was diagnosed with MS, which is a rare manifestation of extramedullary soft tissue masses that may develop as part of AML, myeloproliferative neoplasm (MPN), myelodysplastic syndrome (MDS) or at relapse, especially in patients following allogeneic HSCT.^[26]^ The mechanism and clinical influence of SET-CAN/NUP214 fusion gene in MS is still unknown.

Li MY, et al had reported a MPAL case with the mutation of SET-CAN/NUP214 fusion gene in 2020.^[10]^ MPAL is a rare subtype of acute leukemia, accounting for only 2%-5% of all acute leukemias. The blast cells of MPAL express a complex phenotype of multiple leukemia markers from both the myeloid and lymphoid lineages.^[27]^ MPAL usually comes up with a poor prognosis, particularly in adults. A 29-year-old male patient with a blood cell count of 0.56 × 10^9^/L, and express of CD7, CD34, HLA-DR, CD10, CD19, CD33, CD117, cCD79a, cCD3, was diagnosed B/T MPAL with myeloid lineage expression. The cytogenetic and molecular biological studies showed an abnormality of 46,XY, add(6)t(?1;6)(?p31;p24),del (16)(?q11)[6]/46,XY[4] and SET-CAN/NUP214 fusion gene transcript. After the induction and consolidation treatments the patient achieved complete remission (CR). But the SET-CAN/NUP214 fusion gene was still positive. He then underwent HSCT, but relapsed within 6 months. Then he got the CAR-T cell therapy twice, and alive more than 42 months. This case demonstrated that the efficacy and safety of CAR-T cells infusion for treating recurrent MPAL. And CAR-T may play a contribution to SET-CAN/NUP214 positive acute leukemia.

In general, SET-CAN/NUP214 fusion gene is very rare in adult acute leukemia, more common in T-ALL than in other types of leukemia, and more often in males. The flow cytometry suggested that CD34, CD33, CD13 and CD7 were common markers in SET-CAN/NUP214 positive leukemia, including ALL. The reason why the SET-CAN/NUP214 rearrangement typically induces the expression of myeloid lineage markers such as CD33 and CD13 remains unknown. Since the deletion of SET-CAN fusion gene is so submicroscopic, the wide use of FISH and array is important in newly diagnosed patients, and will detect the del(9)(q34). The chromosomal karyotype may be normal or complex. Whether this gene can cause other critical clinical manifestations remains to be determined. SET-CAN/NUP214 positive patients exhibit marked resistance to induction therapy using corticosteroids and chemotherapy, which may be the result of a combination of various concomitant molecular events and complex genetic aberrations. In terms of survival analysis, transplantation results in a better prognosis than chemotherapy alone. There were reports described the outcome of SET-CAN/NUP214 positive patients was similar to that of SET-CAN/NUP214 negative patients following allo-HSCT, suggesting that HSCT is the most suitable treatment strategy for patients carrying SET-CAN/NUP214 fusion gene. CAR-T is a promising therapy in such patients. It is necessary to detect the SET-CAN/NUP214 fusion gene as a MRD of early recurrence. However, there was a report considered that the SET-CAN fusion did not affect the clinical outcome. Therefore, the mechanism, clinical characteristics, therapy and prognosis of SET-CAN fusion gene in hematological malignancies need more research. Whether the SET-CAN/NUP214 fusion gene may be a useful prognostic indicator in acute leukemia remains to be determined.Author contributionsHZ and JW contributed to the conception of the study.JW and QZ contributed significantly to analysis and manuscript preparation.JW, QZ, and XL performed the data analyses and wrote the manuscript.HZ made the final correction
